# Supplementary material for: MetaRibo-Seq measures translation in microbiomes
Source: Nat Commun. 2020 Jun 29;11:3268. doi: 10.1038/s41467-020-17081-z (PMC7324362; doi:10.1038/s41467-020-17081-z)
Supplement: Supplementary file 10 — Supplementary Data 7 [file 41467_2020_17081_MOESM10_ESM.zip › File2/Confidence_High_Taxonomy/301192_out.krona.html]

Javascript must be enabled to view this page.

members
magnitude
magnitudeUnassigned
count
unassigned
taxon
rank

301192\_out

39

39
superkingdom
2

39
phylum
976

class
200643
39

order
171549
39

37
family
171552

37
genus
838

28
165179

SRS013638\_contig\_number\_10804SRS014415\_contig\_number\_1789SRS017375\_contig\_number\_52SRS018888\_contig\_number\_6566SRS019285\_contig\_number\_contig-100\_402.403SRS019910\_contig\_number\_24175SRS024132\_contig\_number\_16670SRS043841\_contig\_number\_4351SRS046502\_contig\_number\_13745SRS047433\_contig\_number\_6642SRS049712\_contig\_number\_20669SRS050752\_contig\_number\_15622SRS064757\_contig\_number\_7554SRS065397\_contig\_number\_13430SRS077194\_contig\_number\_6280SRS078419\_contig\_number\_28950SRS103987\_contig\_number\_1006SRS104036\_contig\_number\_contig-100\_899.54269SRS1041134\_contig\_number\_contig-100\_1080.81022SRS1041137\_contig\_number\_17328SRS105082\_contig\_number\_2639SRS143876\_contig\_number\_12815SRS144297\_contig\_number\_8868SRS144506\_contig\_number\_42084SRS144714\_contig\_number\_4558SRS146764\_contig\_number\_26095SRS147614\_contig\_number\_4523SRS150029\_contig\_number\_18358
species

species

SRS012989\_contig\_number\_7581SRS013098\_contig\_number\_39322SRS015782\_contig\_number\_41477SRS023914\_contig\_number\_21864SRS044535\_contig\_number\_3632SRS047044\_contig\_number\_17899SRS077991\_contig\_number\_130SRS144603\_contig\_number\_11426
59823
8

1
1263102

SRS016541\_contig\_number\_13165
species

family
815
2


SRS013687\_contig\_number\_15498
816
genus
1
2

1
species

SRS097958\_contig\_number\_8014
818
